# Metabolic surgery for the treatment of type 2 diabetes in obese individuals

**DOI:** 10.1007/s00125-017-4513-y

**Published:** 2017-12-09

**Authors:** David E. Cummings, Francesco Rubino

**Affiliations:** 1 Department of Medicine, Division of Metabolism, Endocrinology and Nutrition, University of Washington, Box 358280 (mail stop 111), Seattle, WA 98195 USA; 2 VA Puget Sound Health Care System, Seattle, WA USA; 3 Department of Surgery, Diabetes and Nutritional Sciences Division, King’s College London and King’s College Hospital, 1st floor James Black Centre, Denmark Hill Campus, 125 Coldharbour Road, London, SE5 9NU UK

**Keywords:** Bariatric surgery, Biliopancreatic diversion, Diabetes Surgery Summit, Gastric bypass, Ghrelin, Glucagon-like peptide-1, Laparoscopic adjustable gastric banding, Metabolic surgery, Review, Vertical sleeve gastrectomy

## Abstract

Several bariatric operations originally designed to promote weight loss have been found to powerfully treat type 2 diabetes, causing remission in most cases, through diverse mechanisms additional to the secondary consequences of weight loss. These observations have prompted consideration of such operations as ‘metabolic surgery’, used expressly to treat diabetes, including among patients who are only mildly obese or merely overweight. Large, long-term observational studies consistently demonstrate that bariatric/metabolic surgery is associated with reductions in all cardiovascular risk factors, actual cardiovascular events, microvascular diabetes complications, cancer and death. Numerous recent randomised clinical trials, directly comparing various surgical vs non-surgical interventions for diabetes, uniformly demonstrate the former to be superior for improvements in all glycaemic variables, as well as other metabolic endpoints. These benefits are similar among individuals with type 2 diabetes and a preoperative BMI of 30–35 kg/m^2^ compared with traditional bariatric surgery patients with a BMI >35 kg/m^2^. The safety profiles of modern laparoscopic bariatric/metabolic operations are similar to those of elective laparoscopic hysterectomy and knee arthroplasty. However, more evidence regarding the risks, benefits and costs of surgery is needed from very long-term (>5 year) randomised clinical trials powered to observe ‘hard’ clinical endpoints following the operations most commonly used today. Given the efficacy, safety and cost-effectiveness of metabolic surgery, the second Diabetes Surgery Summit (DSS-II) consensus conference recently placed surgery squarely within the overall diabetes treatment algorithm, recommending consideration of this approach for patients with inadequately controlled diabetes and a BMI as low as 30 kg/m^2^, or 27.5 kg/m^2^ for Asian individuals. These new guidelines have been formally ratified by 53 leading diabetes and surgery societies worldwide. Given this broad level of endorsement, we feel that the DSS-II recommendations should now replace the outdated National Institutes of Health (NIH) suggestions that have governed bariatric surgery practice and insurance compensation worldwide since 1991.

**Electronic supplementary material:**

The online version of this article (10.1007/s00125-017-4513-y) contains a slideset of the figures for download, which is available to authorised users.

## Introduction

Type 2 diabetes is an expanding pandemic afflicting more than 400 million people, with estimates of 650 million cases by 2040. Despite ever-increasing options for pharmaceutical and lifestyle interventions, including medications recently shown to reduce cardiovascular events, many patients with diabetes fail to achieve glycaemic/metabolic treatment goals designed to reduce micro- and macrovascular complications. In the USA, only 52% of patients with type 2 diabetes maintain HbA_1__c_ <53 mmol/mol (<7%), and only 19% reach this target along with LDL <5.6 mmol/l and blood pressure <130/80 mmHg, as recommended to minimise cardiovascular morbidity and mortality [1]. Implementing more effective strategies to prevent and treat diabetes has become a top priority in 21st century medicine.

Recently, the second Diabetes Surgery Summit (DSS-II), an international consensus conference, developed global guidelines that recommend inclusion of bariatric/metabolic surgery among glucose-lowering interventions for selected patients with type 2 diabetes and obesity [2]. Endorsed thus far by 53 organisations worldwide, including major national diabetes and surgical societies, DSS-II guidelines were incorporated into the ADA Standards of Diabetes Care in 2017 [3]. This new guidance proposes that ‘metabolic surgery’ (involving procedures initially developed to treat obesity and dubbed ‘bariatric surgery’) should be considered as standard diabetes treatment options for appropriate candidates with inadequately controlled type 2 diabetes and a BMI >30 kg/m^2^, or >27.5 kg/m^2^ for Asian individuals. This conclusion is based on biological and clinical rationales. For example, mechanistic studies demonstrate that surgical manipulation of the gastrointestinal tract can exert powerful, beneficial effects on various facets of glucose homeostasis, independent of weight loss [4]. Moreover, a large body of clinical evidence, including numerous randomised clinical trials, documents that surgery improves blood glucose levels more effectively than any lifestyle and/or pharmaceutical intervention, often yielding long-term diabetes remission [5].

Inclusion of surgery among standard diabetes therapies represents a significant step forward in diabetes care and research. The mechanisms of metabolic surgery, albeit incompletely understood, underscore important roles for the gut in glucose homeostasis. Elucidating these mechanisms provides opportunities to clarify type 2 diabetes pathogenesis, potentially identifying targets for novel pharmacotherapeutics.

Leveraging insights provided by metabolic surgery, however, requires addressing practical and conceptual barriers, including widespread misconceptions about bariatric surgery. Despite compelling evidence of safety, efficacy and cost-effectiveness, using surgery as a diabetes intervention remained controversial until very recently.

Herein we review evidence regarding the effects of metabolic surgery in patients with obesity and type 2 diabetes, discussing the clinical, biological and economic rationales that support expanding its use as part of modern multidisciplinary approaches to diabetes care.

## Biological rationale for considering bariatric/metabolic surgery to treat type 2 diabetes

It has become clear that certain operations initially designed to promote weight loss also powerfully improve glucose homeostasis, leading to type 2 diabetes remission in most cases, especially after procedures with intestinal bypass components [5]. Although approximately one-third of patients who initially achieve diabetes remission after Roux-en-Y gastric bypass (RYGB) later experience relapse, the median disease-free period for these individuals is 8.3 years [6]. Given the known benefits of tight vs standard glycaemic control in early diabetes on long-term cardiovascular disease (‘legacy effect’) [7, 8], even among people whose diabetes relapses several years after metabolic surgery, it is possible that this disease-free interval will also yield long-term cardiovascular benefits in relapsed individuals, especially in cases where initial diabetes duration is relatively short. Although this has not yet been proven in randomised clinical trials, very large, rigorously matched, non-randomised studies have shown that metabolic surgery is associated with long-term reductions in all cardiovascular risk factors, actual cardiovascular events, cancer and death [5, 9–12].

It is also clear that many diabetes-associated benefits of intestinal bypass operations, such as RYGB, result not only from known effects of weight loss on glucose homeostasis but also from diverse weight-independent glucose-lowering mechanisms. Several large bodies of evidence demonstrate this [13]. First, diabetes commonly remits very fast after surgery, before significant weight loss. Second, for a given amount of weight loss achieved with intestinal bypass surgery, larger improvements in glucose homeostasis and diabetes occur than with equivalent weight loss achieved by dieting, exercise or purely gastric-restrictive operations. Third, there is an inconsistent relationship between the amount of weight lost after intestinal bypass operations and the degree of diabetes remission, prevention and relapse after initial remission, as well as with rates of improvement in hard outcomes, such as heart attacks, strokes, cancer and death. Fourth, experimental operations and devices that replicate some of the intestinal physiology of metabolic operations, such as RYGB, without compromising gastric capacity, can powerfully improve or eliminate type 2 diabetes with little or no weight loss, disengaging the weight-reducing and glucose-lowering effects of surgery. Finally, rare but illuminating cases of profound, late-onset hyperinsulinaemic hypoglycaemia (occurring 1–26 years postoperatively, typically at 2–4 years), sometimes requiring pancreatectomy, suggest long-term post-surgical stimulation of beta cell function and, possibly, mass. This latter point demonstrates that, occasionally, the effectiveness of surgery for the treatment of diabetes can be ‘too powerful’, something that would never occur with non-surgical weight loss.

A partial list of mechanisms mediating weight-independent glucose-lowering effects of gastrointestinal surgery is shown in the text box below. Although many of these specific mechanisms have only been demonstrated thus far in animals, compelling evidence indicates that metabolic surgery engages weight-independent glucose-lowering processes in humans [4]. This has important clinical implications because, although individuals with a lower BMI lose less weight postoperatively than do the severely obese, they still experience these weight-independent glucose-lowering effects. Accordingly, the benefits of bariatric/metabolic surgery for type 2 diabetes appear to be similar among people with a preoperative BMI <35 kg/m^2^ to those with a BMI ≥35 kg/m^2^ [14], the traditional cut-off for bariatric surgery in patients with diabetes (see below). Admittedly, evidence for this assertion is limited for patients with a preoperative BMI <30 kg/m^2^.
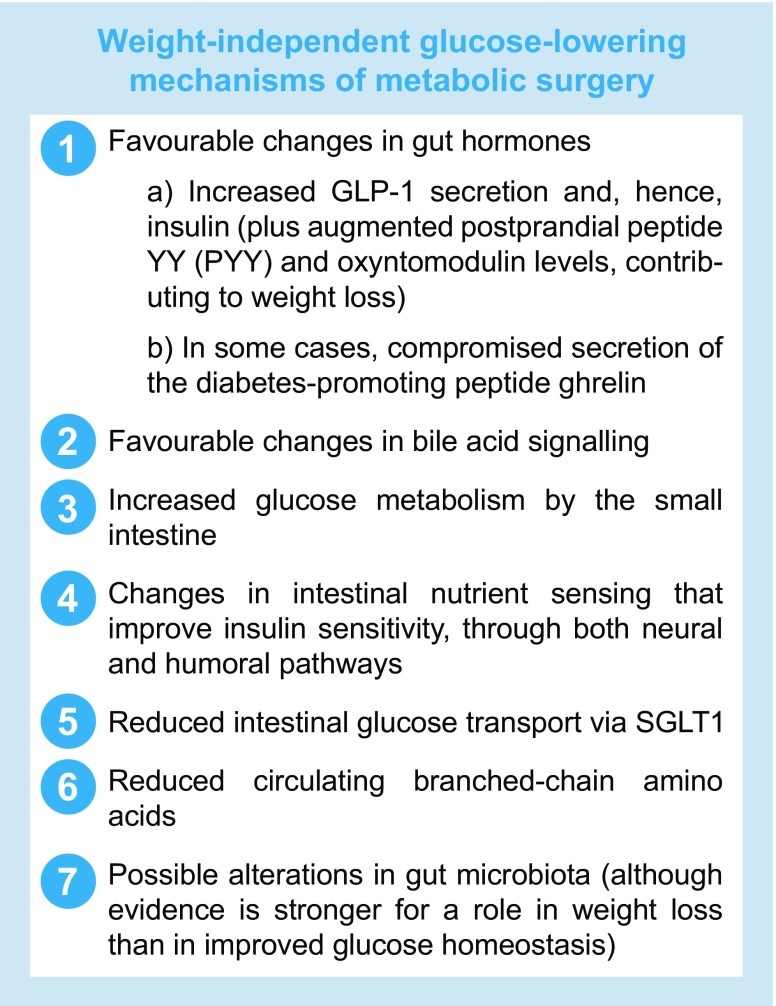


The existence of weight-independent glucose-lowering mechanisms activated by traditional ‘bariatric’ operations, along with the profound clinical benefits of these procedures on type 2 diabetes (including among non-severely obese individuals), has prompted increasing consideration of surgery to treat diabetes per se as the primary intent, even among patients who are only mildly obese or merely overweight [15]. Hence, the term ‘metabolic surgery’ is becoming increasingly popular, and virtually every bariatric surgery society in the world has changed its name within the past several years to include the word ‘metabolic’.

## Clinical rationale for considering metabolic surgery to treat type 2 diabetes

Several large, long-term observational studies have uniformly shown that, compared with people receiving non-surgical care for obesity and diabetes, those who elect to undergo bariatric/metabolic surgery demonstrate greater improvements in body weight, glycaemic control, diabetes remission, other cardiovascular risk factors, microvascular complications, heart attacks, strokes, cancer and death [5, 9–12]. Nine studies show reductions in all-cause mortality among patients who have undergone bariatric/metabolic surgery [5, 11, 16, 17], including a remarkable 92% decrease in diabetes-related deaths [17], and none have failed to observe this. Because these studies are not randomised clinical trials, however, potential unmeasured confounders are a concern. Perhaps people who elect to undergo a major intervention, such as surgery, are more motivated to maintain other healthy behaviours, such as changing their diet, exercising, ceasing smoking, seeing their doctors, taking medications, etc.

To address these concerns about unmeasured confounders in non-randomised studies, 11 recent randomised clinical trials have directly compared surgical vs non-surgical diabetes interventions. A DSS-II meta-analysis of these trials found almost universal results among them [2, 5]. Compared with a wide variety of medical/lifestyle interventions, the four most commonly performed bariatric/metabolic operations (see below) consistently yielded superior improvements in body weight, all glycaemic measures (diabetes remission, glycaemic control, diabetes medication use, etc.), HDL and triacylglycerol levels, the metabolic syndrome, quality of life and overall medication use. These benefits were associated with acceptable complications and no surgical deaths to date (including follow-up of 1–5 years), among the 1050 surgical participants included. These findings represent nearly unanimous level 1a evidence from the meta-analysis of randomised clinical trials, including data on many patients with a preoperative BMI <35 kg/m^2^. Surgical patients also tended to experience greater improvements in LDL levels and hypertension than did non-surgical participants in these randomised clinical trials, although these differences were not as profound or consistent as the results for diabetes-related variables [2, 5, 18].

## Evidence for use of surgery to treat type 2 diabetes in patients with a BMI less than vs greater than 35 kg/m^2^

Traditionally, bariatric surgery is only permitted in people with type 2 diabetes if their BMI is ≥35 kg/m^2^. This threshold is increasingly being challenged when considering metabolic surgery for diabetes. A recent meta-analysis of all published studies reporting on diabetes remission after bariatric/metabolic surgery (including 94,579 surgical patients) showed that remission rates were equivalent in the 60 investigations in which mean baseline BMI of the study cohorts was ≥35 kg/m^2^ and the 34 studies including participants with mean baseline BMI <35 kg/m^2^ (71% vs 72%, respectively) [19].

**Fig. 1:**
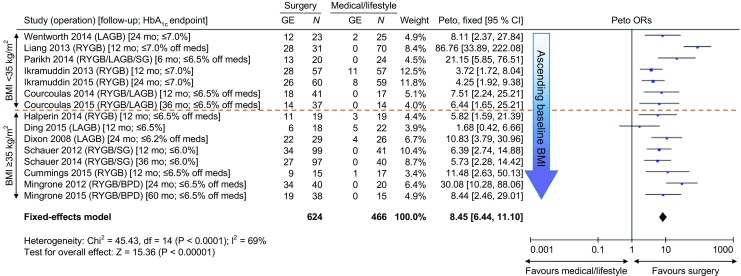
Odds of diabetes remission or glycaemic control in all 11 randomised clinical trials of surgery vs medical/lifestyle care for type 2 diabetes. Forest plot of Peto ORs of primary outcomes (main glycaemic endpoints [GE], i.e. either diabetes remission or glycaemic control, depending on the trial) from each of the 11 published randomised clinical trials directly comparing bariatric/metabolic surgery vs medical/lifestyle treatments for diabetes. Data are arranged in order of ascending mean baseline BMI of each study group. The orange dotted line demarcates trials performed on cohorts with an average starting BMI either <35 kg/m^2^ or ≥35 kg/m^2^. Column 1 depicts study duration and HbA_1c_ endpoint thresholds (in square brackets). Here, ‘off meds’ refers to a threshold achieved off all diabetes medicines, whereas otherwise the endpoints represent thresholds attained with or without such agents. ORs (shown with 95% CI) >1 indicate a positive effect of surgery compared with medical/lifestyle treatment. The pooled Peto OR (95% CI) for all data was calculated using a fixed-effects model. mo, months; SG, sleeve gastrectomy. © 2016 by the ADA [14]. Adapted with permission from the ADA

Similarly, the previously mentioned meta-analysis of 11 existing randomised clinical trials directly comparing various surgical vs non-surgical approaches to type 2 diabetes treatment found that the degree to which surgery out-performed medical/lifestyle interventions for diabetes remission and/or glycaemic control was equivalent among the five randomised clinical trials in which mean baseline BMI of the study cohorts was <35 kg/m^2^ compared with the six randomised clinical trials with mean participant baseline BMI ≥35 kg/m^2^ (Fig. 1) [14].

Furthermore, according to a comprehensive comparative effectiveness analysis by the Agency for Healthcare Research and Quality (AHRQ), the safety of bariatric/metabolic surgery is at least as good for patients with a preoperative BMI <35 kg/m^2^ as for those with baseline BMI ≥35 kg/m^2^ [20].

The similar efficacy and safety of metabolic surgery to treat type 2 diabetes in patients with a preoperative BMI above vs below 35 kg/m^2^ indicates that this arbitrary threshold, established 26 years ago by the National Institutes of Health (NIH) to determine surgical eligibility, is not well supported by extant evidence [21].

## Safety of metabolic surgery for type 2 diabetes

Bariatric/metabolic surgery has become progressively safer over the past two decades, largely owing to the refinement of minimally invasive techniques. The vast majority of operations are now performed laparoscopically, with surgical mortality rates ten times less than for equivalent open operations [22]. Perioperative mortality rates from bariatric/metabolic surgery are less than those from laparoscopic cholecystectomy or appendectomy, and similar to elective laparoscopic hysterectomy and knee arthroplasty [23]. The perioperative complication rate for laparoscopic RYGB in a recent US National Registry is 3.4%, which is less than that for laparoscopic hysterectomy, cholecystectomy or appendectomy [23].

All operations involve risk, and the most common long-term complications of bariatric/metabolic surgery are listed in the text box below [5]. Among the most frequent of these, iron deficiency can be chronic and sometimes requires repeated iron infusions. In addition, the incidence of less frequent but very serious adverse outcomes, for example suicide, accidental injuries and alcohol abuse, is substantially higher after some operations, with relative risk rates increasing by two- to threefold [5]. There is also considerable concern about long-term risks after certain procedures (especially those involving intestinal bypasses) on metabolic bone disease, osteoporosis and fracture rates. Definitive evidence regarding these hazards is incomplete.
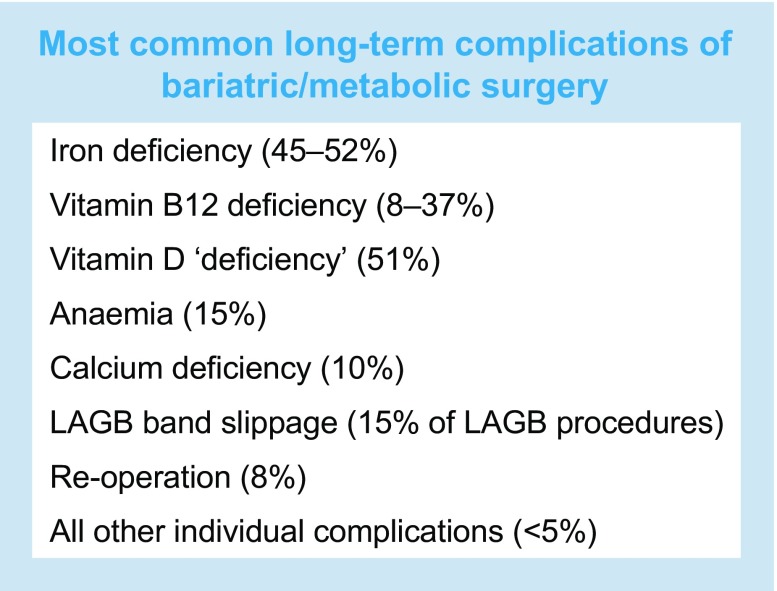


Importantly, these potential complications must be weighed against possible adverse consequences of not having surgery, such as typically remaining obese, continuing to have diabetes and taking glucose-lowering medications (which can cause hypoglycaemia, weight gain, and other problems). As stated above, all nine existing published studies of overall long-term mortality among patients who have undergone bariatric/metabolic surgery have reported that death rates are reduced compared with those in non-surgical controls [5, 11, 16, 17]. The aforementioned comprehensive AHRQ review of surgical vs non-surgical approaches to metabolic conditions, such as diabetes, concluded that adverse events of surgery were relatively low and that most surgical complications were minor and tended not to require major intervention [20].

## Economic rationale for considering metabolic surgery to treat type 2 diabetes

Although only some studies show bariatric/metabolic surgery to be outright cost-saving, all studies find it cost-effective, with a price per quality-adjusted life-year (QALY) of ~US$5000–10,000, well below the commonly accepted standard of US$50,000/QALY for affordable healthcare interventions [24, 25]. The cost-effectiveness of surgery is greater among people with diabetes than in those without it [25]. By comparison, as examples of non-surgical diabetes treatments, intensive medicinal glycaemic and lipid control cost US$41,384/QALY and US$51,889/QALY, respectively [26]. An important caveat is that most economic analyses of bariatric/metabolic surgery derive from modelling studies, rather than direct measurements within randomised clinical trials.

The time to return-on-investment for surgery varies widely among studies, from only a few years to as many as 9 years (or not observed within the period of follow-up). However, it is likely that the cost of bariatric/metabolic operations is fully repaid at some point after surgery if patients live long enough, from savings on medications not taken, hospitalisations avoided, complications not suffered, etc. Hence, insurance plans that typically retain patients for a long time (e.g. the Veterans Health Administration) should ultimately recoup the entire cost of surgery. Moreover, if all insurance plans covered bariatric/metabolic surgery, long-term healthcare costs for the entire system would decrease. Similar benefits would be observed in public healthcare systems, such as the UK National Health Service (NHS).

## Choosing the appropriate operation for type 2 diabetes

Bariatric/metabolic operations that are currently in clinical use include RYGB (48% of bariatric/metabolic surgery worldwide), vertical sleeve gastrectomy (VSG, 42%), laparoscopic adjustable gastric banding (LAGB, 8%) and biliopancreatic diversion (BPD, 2%). The order of effectiveness for weight loss and diabetes improvement is BPD>RYGB>VSG>LAGB. The opposite order applies for safety.

By far, the most commonly used operations are RYGB and VSG, with VSG having overtaken RYGB in many nations. However, RYGB is more effective against diabetes and is considered by many as the gold standard operation for patients with this disease.

Novel bariatric/metabolic operations and devices are presently under development, including: (1) proximal intestinal bypass procedures for type 2 diabetes (e.g., duodenal-jejunal bypass surgery, endoscopically implanted endoluminal sleeves and endoscopic duodenal mucosal resurfacing); (2) ileal interposition surgery to enhance distal intestinal nutrient stimulation of L-cell peptide secretion; and (3) endoscopic techniques to reduce gastric volume for weight loss (e.g. new-generation gastric balloons, gastric plication and gastric electrical stimulation). Most of these approaches are currently used primarily in clinical trials.

## Evidence gaps in metabolic surgery research

Although the mechanistic and clinical databases supporting a role for metabolic surgery in the overall diabetes treatment algorithm have burgeoned in recent years, including highly consistent level 1a evidence from randomised clinical trials directly comparing surgical vs non-surgical approaches, many key domains warrant additional research. Prominent among these are the following:

1. Long-term (>5 year) results from randomised clinical trials regarding durability of glycaemic improvements are lacking. Efforts are underway to redress this gap, such as the Alliance of Randomized Trials of Medicine vs Metabolic Surgery in Type 2 Diabetes (ARMMS-T2D; ClinicalTrials.gov registration no. NCT02328599) consortium randomised clinical trial.
2. Longer-term data (>10 years) are needed on efficacy and safety using non-randomised studies examining the operations currently performed. Although the superlative Swedish Obese Subjects (SOS) study database [9] is very large and extremely long term (and the most cited study of this nature in this field), it is primarily derived from observations in people with vertical-banded gastroplasty, which has not been used for decades, and LAGB, which is now seldom performed. Comparatively few patients underwent RYGB in this study and none had VSG, yet these two operations constitute 90% of bariatric/metabolic surgery currently performed.
3. Level 1 evidence is needed from randomised clinical trials sufficiently powered and of long enough duration to measure hard outcomes, such as microvascular and/or macrovascular events, cancer, death, etc.
4. More evidence is warranted regarding the long-term risks after intestinal bypass operations for metabolic bone disease, osteoporosis and fractures, especially from operations most commonly performed today.
5. We need true cost-effectiveness data from large randomised clinical trials, rather than just modelling methodologies.
6. Now that modern diabetes medications such as glucagon-like peptide-1 (GLP-1) agonists and sodium–glucose cotransporter (SGLT) 2 inhibitors have been shown to confer cardiovascular protection in large trials, these newer agents need to be directly compared against metabolic surgery in randomised clinical trials with ‘hard’ endpoints.
7. Level 1 evidence is needed regarding the long-term (>5 year) rates of weight regain and diabetes recurrence after VSG, which is now the most commonly performed bariatric/metabolic operation in many nations.
8. It is likely that the combination of metabolic surgery and intensive medical/lifestyle treatment is the most effective strategy for diabetes control, and further research on the details of combining these approaches is warranted.

## New international guidelines for metabolic surgery to treat type 2 diabetes from the DSS-II

In 2007, the first Diabetes Surgery Summit encouraged greater research on mechanisms of metabolic surgery as well as randomised clinical trials to define its role in type 2 diabetes management [27, 28]. This helped generate funding to develop the evidence highlighted here. Based on this evidence, in 2015/2016 a panel of 48 international diabetes experts, representing numerous major worldwide diabetes societies, utilised a formal Delphi-like consensus-development process to generate new guidelines regarding the use and study of metabolic surgery for type 2 diabetes. Their recommendations were finalized at the DSS-II and, subsequently, codified in numerous related publications.

**Fig. 2:**
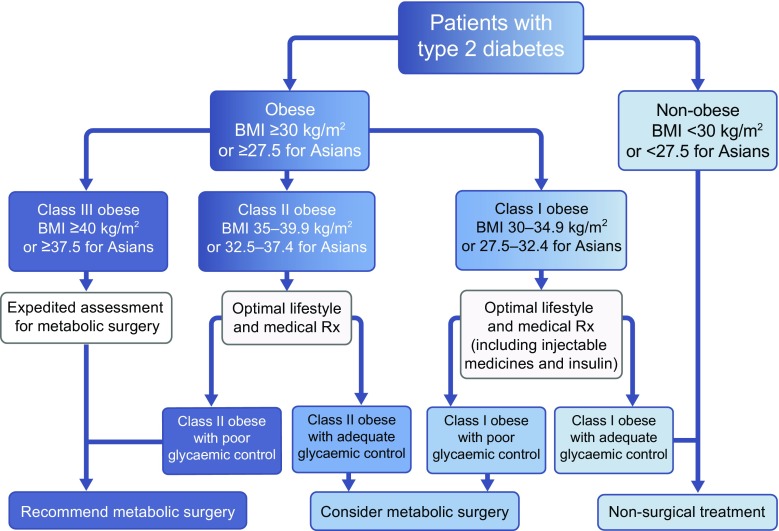
DSS-II: surgery in the type 2 diabetes treatment algorithm. Algorithm for the treatment of type 2 diabetes, including the option of bariatric/metabolic surgery, as recommended by DSS-II voting delegates. Rx, treatment. © 2016 by the ADA [2]. Adapted with permission from the ADA

The 32 new DSS-II consensus statements and guidelines identify metabolic surgery as a standard option in the type 2 diabetes treatment algorithm (Fig. 2) [2]. With very high consensus, the delegates recommended that surgery should be considered to treat inadequately controlled type 2 diabetes in people with a BMI as low as 30 kg/m^2^ (vs the prior NIH cut-off of 35 kg/m^2^), or as low as 27.5 kg/m^2^ in Asian patients. Although this change in BMI threshold is a modest numerical modification, it encompasses a very large number of people. In the USA, ~43% of individuals with diabetes have a BMI of 30–35 kg/m^2^ [1] and, worldwide, the vast majority of people with diabetes have a BMI <35 kg/m^2^, including >98% of East Asian individuals [29].

The DSS-II final recommendations have been formally ratified by 52 international societies thus far, including the ADA, the International Diabetes Federation (IDF), the Chinese Diabetes Society, Diabetes India, the European Association for the Study of Obesity (EASO), the Endocrine Society, the American Association of Clinical Endocrinologists (AACE), The Obesity Society and the American Gastroenterological Association (AGA), as well as the national diabetes organisations of many European and South American countries [2]. Hopefully, with this high degree of vetting and worldwide endorsement, these new guidelines will finally replace the seriously outdated 1991 NIH recommendations that have governed global practice and insurance compensation of bariatric surgery for more than a quarter of a century [21].

## Electronic supplementary material

ESM Downloadable slideset (PPTX 479 kb)

## Abbreviations

AHRQ: Agency for Healthcare Research and Quality
BPD: Biliopancreatic diversion
DSS-II: Second Diabetes Surgery Summit
GLP-1: Glucagon-like peptide-1
LAGB: Laparoscopic adjustable gastric banding
NIH: National Institutes of Health
QALY: Quality-adjusted life-year
RYGB: Roux-en-Y gastric bypass
SGLT: Sodium–glucose cotransporter
VSG: Vertical sleeve gastrectomy
