# Supplementary material for: Metabolic surgery for the treatment of type 2 diabetes in obese individuals
Source: Diabetologia. 2017 Dec 9;61(2):257–64. doi: 10.1007/s00125-017-4513-y (PMC6448954; doi:10.1007/s00125-017-4513-y)
Supplement: Supplementary file 1 — (PPTX 479 kb) [file 125_2017_4513_MOESM1_ESM.pptx]

## Slide 1
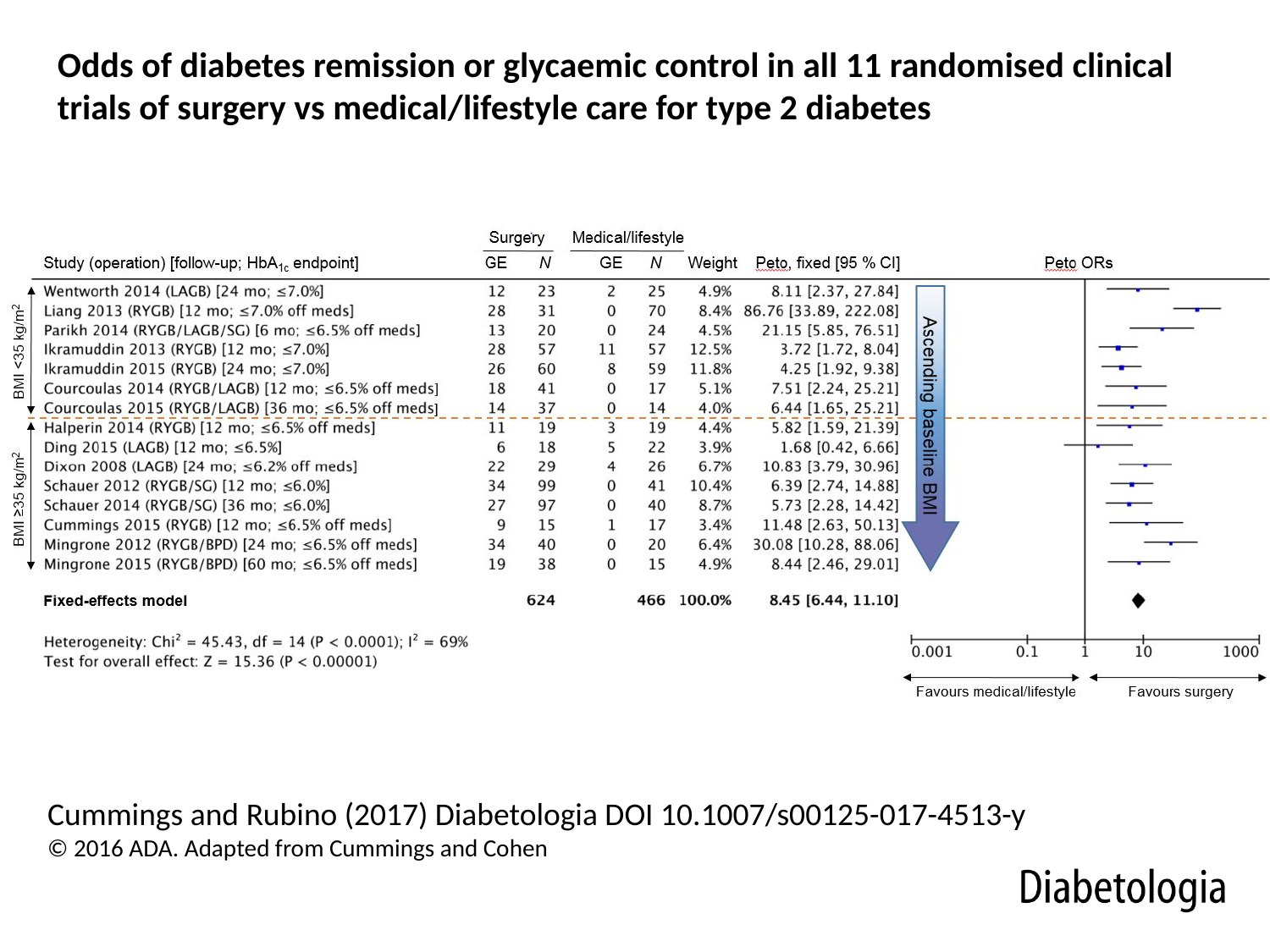

Odds of diabetes remission or glycaemic control in all 11 randomised clinical trials of surgery vs medical/lifestyle care for type 2 diabetes
Cummings and Rubino (2017) Diabetologia DOI 10.1007/s00125-017-4513-y
© 2016 ADA. Adapted from Cummings and Cohen

## Slide 2
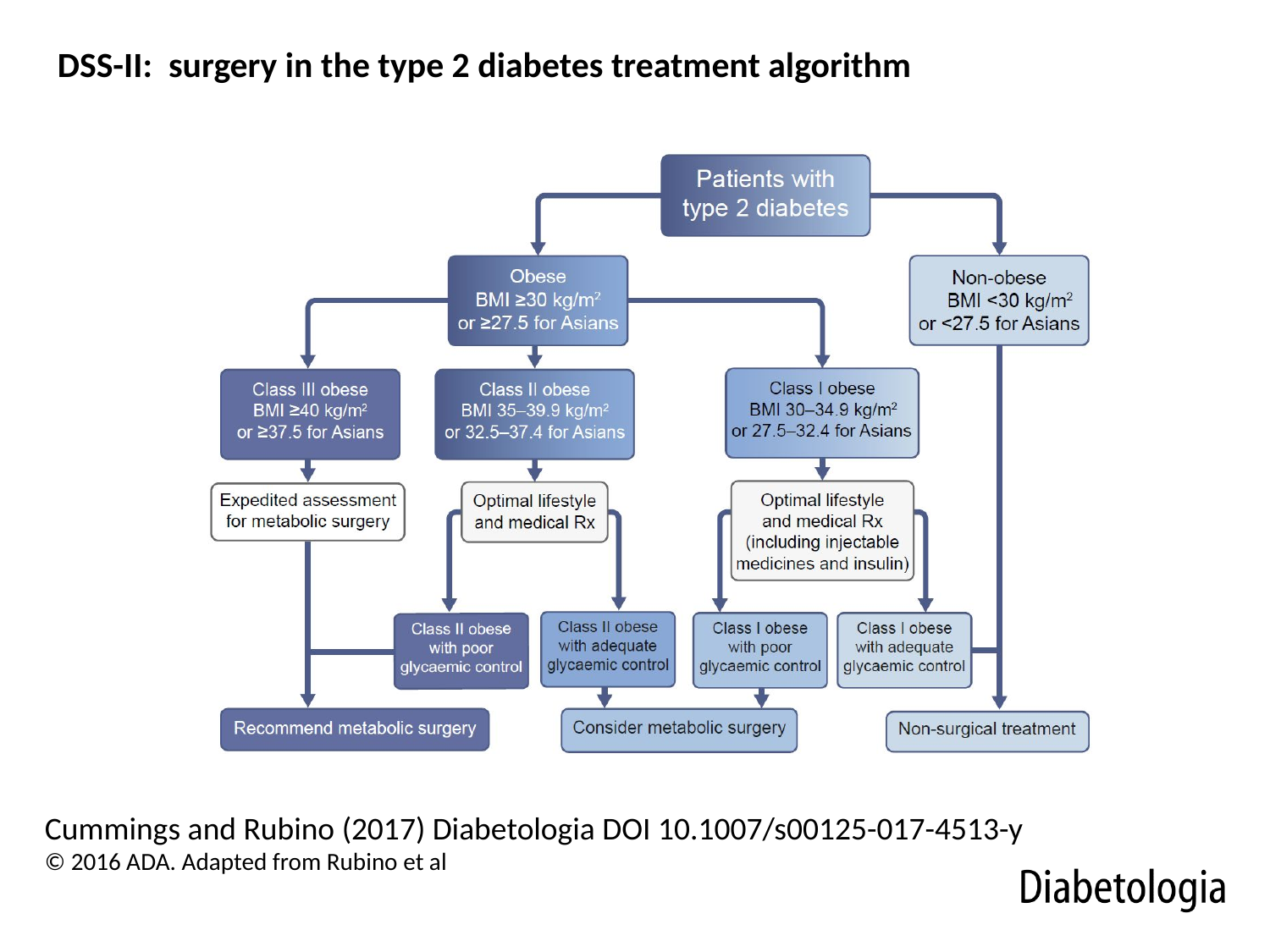

DSS-II: surgery in the type 2 diabetes treatment algorithm
Cummings and Rubino (2017) Diabetologia DOI 10.1007/s00125-017-4513-y
© 2016 ADA. Adapted from Rubino et al
